# Multicentre validation of the PRECISE scoring system for prostate MRI during active surveillance

**DOI:** 10.1007/s00330-026-12570-z

**Published:** 2026-05-04

**Authors:** Francesco Giganti, Riccardo Leni, Vinayak Wagaskar, Giorgio Gandaglia, Tristan Barrett, Valeria Panebianco, Francesco Sanguedolce, Erik JRJ van der Hoeven, Sangeet Ghai, Jeremy Grummet, Jasmin Gmeiner, Jan Philipp Radtke, Ryan D. Ward, Ronaldo Hueb Baroni, Francesco Porpiglia, Juan Gomez Rivas, Fabio Zattoni, Raphaële Renard-Penna, Claudia Kesch, Marco Gatti, Nicola Schieda, Guillaume Ploussard, Sara Lewis, Giorgio Brembilla, Christof Kastner, Emanuele Messina, Ash Tewari, Caroline M. Moore, Massimo Valerio, Armando Stabile, Veeru Kasivisvanathan

**Affiliations:** 1 Division of Surgery and Interventional Science, University College London, London, UK; 2 Department of Radiology, University College London Hospitals NHS Foundation Trust, London, UK; 3 Division of Oncology/Unit of Urology, Soldera Prostate Cancer Lab, URI, IRCCS San Raffaele Scientific Institute, Milan, Italy; 4 Department of Urology, Icahn School of Medicine at Mount Sinai Hospital, New York, NY, USA; 5 Department of Radiology, Addenbrooke’s Hospital and University of Cambridge, Cambridge, UK; 6 Department of Radiological Sciences, Oncology and Pathology, Sapienza University/Policlinico Umberto I, Rome, Italy; 7 Department of Urology, Fundació Puigvert, Barcelona, Spain; 8 Department of Medicine, Surgery and Pharmacy, University of Sassari, Sassari, Italy; 9 Department of Radiology, St Antonius Hospital, Utrecht, The Netherlands; 10 Joint Department of Medical Imaging, University Health Network–Mt Sinai Hospital–Women’s College Hospital, University of Toronto, Toronto, ON, Canada; 11 Department of Surgery, Alfred Health, School of Translational Medicine, Monash University, Melbourne, Australia; 12 Department of Biomedical Imaging and Image-guided Therapy, Medical University of Vienna, Vienna, Austria; 13 Department of Urology, Medical Faculty, Heinrich-Heine University Düsseldorf, Düsseldorf, Germany; 14 Department of Radiology, German Cancer Research Centre, Heidelberg, Germany; 15 Abdominal Imaging Section, Diagnostics Institute, Cleveland Clinic, Cleveland, OH, USA; 16 Imaging Section, Hospital Israelita Albert Einstein, Sao Paulo, Brazil; 17 Division of Urology, Department of Oncology, San Luigi Gonzaga Hospital, University of Turin, Turin, Italy; 18 Department of Urology, Health Research Institute, Hospital Clinico San Carlos, Madrid, Spain; 19 Department of Surgery, Oncology, and Gastroenterology, Urology Clinic, University of Padua, Padua, Italy; 20 Department of Imaging, Hôpital Pitié Salpêtrière APHP–Sorbonne University, Paris, France; 21 Department of Urology, University of Duisburg-Essen and German Cancer Consortium (DKTK), University Hospital Essen, Essen, Germany; 22 Division of Radiology, Department of Surgical Sciences, Molinette Hospital, Città della Salute e della Scienza and University of Turin, Turin, Italy; 23 Department of Radiology, The University of Ottawa, Ottawa, ON, Canada; 24 La Croix du Sud Hospital, Department of Urology, Quint-Fonsegrives, France; 25 Department of Radiology, Icahn School of Medicine at Mount Sinai Hospital, New York, NY, USA; 26 Department of Radiology, IRCCS San Raffaele Scientific Institute, Vita-Salute San Raffaele University, Milan, Italy; 27 Department of Urology, Addenbrooke’s Hospital and University of Cambridge, Cambridge, UK; 28 Department of Urology, University College London Hospitals NHS Foundation Trust, London, UK; 29 Department of Urology, Lausanne University Hospital, Lausanne, Switzerland; 30 University College London Hospitals, London, UK; 31 University College London, London, UK; 32 Icahn School of Medicine at Mount Sinai Hospital, New York, NY, USA; 33 IRCCS San Raffaele Scientific Institute, Milan, Italy; 34 Addenbrooke’s Hospital and University of Cambridge, Cambridge, UK; 35 Sapienza University/Policlinico Umberto I, Rome, Italy; 36 Lausanne University Hospital, Lausanne, Switzerland; 37 Fundació Puigvert, Barcelona, Spain; 38 St Antonius Hospital, Utrecht, The Netherlands; 39 University Health Network, Toronto, ON, Canada; 40 Monash University, Melbourne, Australia; 41 Medical University of Vienna, Vienna, Austria; 42 Heinrich-Heine-University Düsseldorf, Düsseldorf, Germany; 43 Cleveland Clinic, Cleveland, OH, USA; 44 Hospital Israelita Albert Einstein, Sao Paulo, Brazil; 45 IRCCS Candiolo Cancer Institute, Turin, Italy; 46 Health Research Institute, Hospital Clinico San Carlos, Madrid, Spain; 47 University of Padova, Padova, Italy; 48 University Hospital Essen, Essen, Germany

**Keywords:** Prostatic neoplasms, Magnetic resonance imaging, Prostate biopsy

## Abstract

**Objectives:**

The Prostate Cancer Radiological Estimation of Change in Sequential Evaluation (PRECISE) recommendations assess radiological change in serial MRI during active surveillance (AS) for prostate cancer. PRECISE 1–2 indicates radiological regression, PRECISE 3 stability, and PRECISE 4–5 progression. Our aim was to test the PRECISE score as a predictive tool for disease progression in a multicentre international setting.

**Materials and methods:**

This is a retrospective study in which we collected data from 22 international centres from December 2005 to July 2022, applying two entry criteria: (1) at least two scans (baseline and follow-up); (2) at least two biopsies (baseline and follow-up, the latter after the second scan). Local radiologists reported scans according to PRECISE. Sensitivity, specificity, positive predictive value (PPV) and negative predictive value (NPV) according to different biopsy thresholds and definitions of progression were calculated.

**Results:**

A total of 1667 patients were included. Median follow-up was 4 years (IQR: 2.1–6.3). A total of 1248 (75%) patients underwent two MRIs and immediate subsequent biopsy, 300 (24%) of which had biopsy progression to Grade Group ≥ 2 and 77 (6%) to Grade Group ≥ 3. Patients with PRECISE 4–5 had 4.53-fold increased odds (95% CI: 3.37–6.12; *p* < 0.001) of biopsy progression compared to PRECISE 1–3. Using a PRECISE ≥ 4 cutoff to perform follow-up biopsies, overall sensitivity, specificity, PPV and NPV were 57%, 79%, 46%, and 85% for the first follow-up scan.

**Conclusion:**

The PRECISE recommendations could lead to timely identification of patients on AS who progress on MRI, prompting re-biopsy or treatment, and safe reduction of repeat biopsies for those with stable MRI and prostate-specific antigen kinetics.

**Key Points:**

***Question*** *Can the PRECISE scoring system for monitoring prostate cancer lesions on active surveillance on MRI predict disease progression and avoid unnecessary biopsies?*

***Findings*** *The PRECISE score effectively predicts prostate cancer progression, with PRECISE 4–5 (progression) scores indicating 4.53-fold increased odds of biopsy progression compared to PRECISE 1–3 (regression/stability).*

***Clinical relevance*** *This study validates PRECISE as a tool for managing patients on active surveillance. It can help clinicians identify those needing timely re-biopsy or treatment, while reducing unnecessary biopsies in patients with stable disease on imaging and PSA kinetics.*

**Graphical Abstract:**

**Figure:**
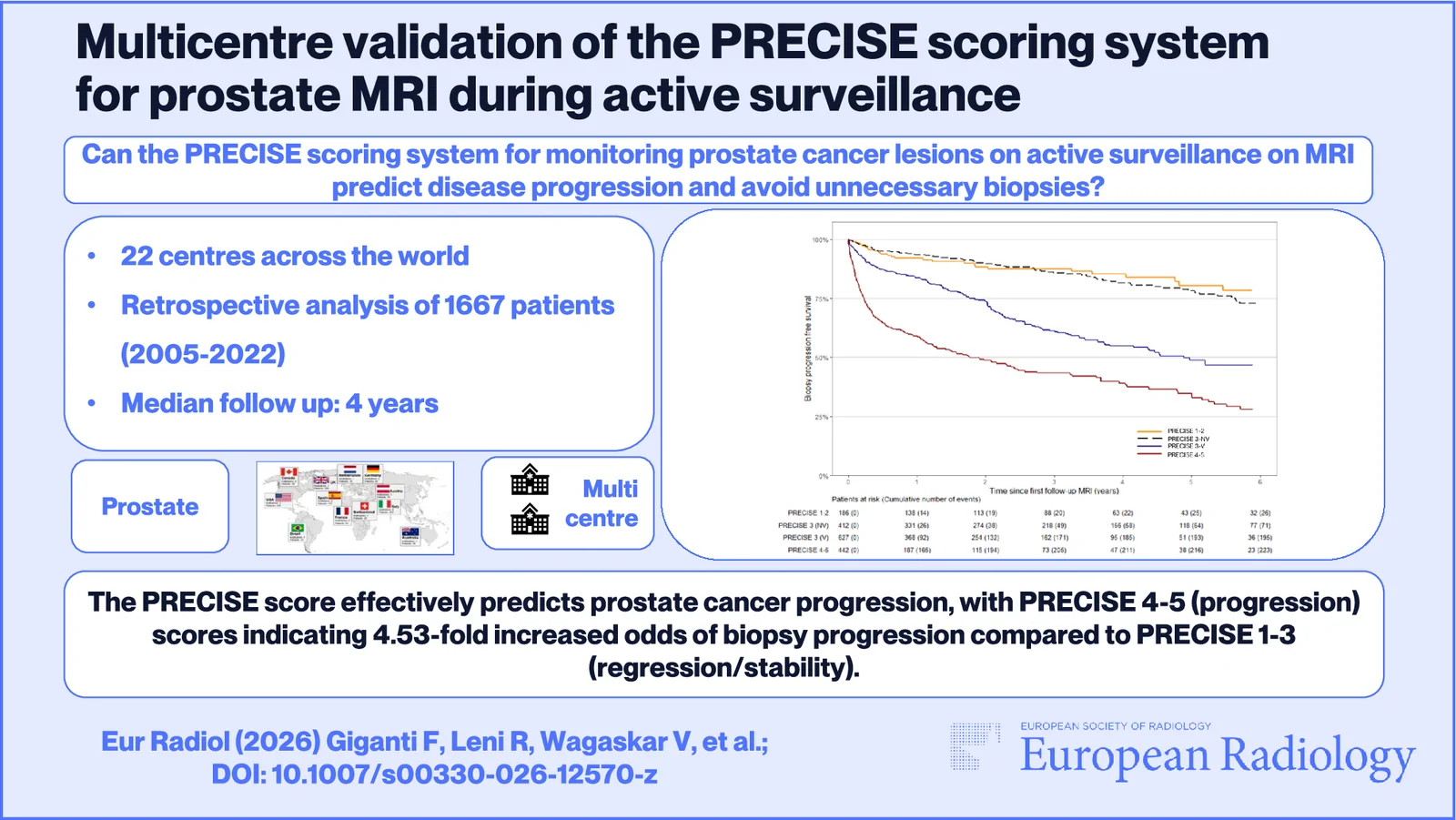

## Introduction

Patients with low and favourable intermediate-risk prostate cancer are eligible for active surveillance (AS), which aims to reduce over-treatment and treatment-related side effects without losing the window of curability. Such an approach involves close monitoring with the intent to intervene for disease progression [1]. Several factors, including the clinical stage, prostate-specific antigen (PSA) level, and biopsy results (which include the Gleason score and an estimate of cancer burden), are considered when enrolling a patient in AS [2]. There are different AS protocols across the world, and they usually consist of a combination of serial PSA tests, digital rectal examination, prostate biopsies, and magnetic resonance imaging (MRI) [2–4].

Interest in the use of MRI during AS has grown thanks to its high negative predictive value for clinically significant disease and because it may also increase patient compliance with AS [5–7]. It has become a crucial tool for improving patient selection for AS and can be useful in increasing the accuracy of targeted biopsy, identifying both patients who would benefit most and those who could safely defer or omit further biopsy, as shown in the ASIST trial where patients randomised to the MRI (targeted + systematic) arm had a reduced rate of failure of AS at 2 years compared to those randomised to TRUS biopsy (19% vs 35%, *p* = 0.017) [8].

The ability to effectively use MRI is crucial for directing management choices regarding biopsy or treatment for patients on AS. The Prostate Cancer Radiological Estimation of Change in Sequential Evaluation (PRECISE) guidelines were developed in 2016 to standardise and streamline the reporting of serial prostate MRI during AS and have been recently updated (PRECISE v2) [9, 10]. They include a scale ranging from 1 to 5 to indicate the possibility of radiological change over time. On this scale, PRECISE 1–2 indicates radiological regression, PRECISE 3 signifies radiological stability (divided into 3-visible and 3-NonVisible in version 2), and PRECISE 4–5 suggests radiological progression (within or outside of the prostate, respectively). The aim of our study was to evaluate the utility of this scoring system in an international multicentre setting for the first time.

## Methods

This is a multicentre, retrospective, observational study of men undergoing serial MRI during AS, assessing the degree of radiological change over time using the PRECISE criteria. No commercial entity was involved in the study, which was funded by the Prostate Cancer Foundation and the Cris Cancer Foundation. The funders had no role in the study development, data analysis or interpretation, or manuscript preparation.

### Study design and participants

Different participating centres across the world were invited to join a pilot collaborative task force based on their robust and well-established use of prostate MRI as part of AS. The scans included in the study ranged from December 2005 to July 2022. All clinical records and MR images were reviewed locally and managed as per the standard of care by the treating team.

The inclusion criteria were: (1) patients on AS for localised prostate cancer diagnosed on biopsy who underwent a pre- or post-biopsy MRI and at least one subsequent follow-up MRI scan; (2) Gleason Grade Group (GG) 1 or GG 2 at entry biopsy; (3) an additional prostate biopsy (‘per protocol’ or ‘for cause’, which means due to changes in clinical or imaging features) after a specific follow-up MRI scan.

The exclusion criteria were: (1) clinical T3 disease; (2) unequivocal radiological T3 disease; (3) any previous prostate treatment; (4) poor image quality MRI (e.g., due to significant artefacts from metalwork or other causes assessed independently by each local radiologist, corresponding to PRECISE ‘X’ in PRECISE v2).

The reference standard was histopathology from a biopsy.

### Procedures

All scans were scored using the Prostate Imaging and Reporting Data System (PI-RADS) v. 2.1 system. MRI was acquired on either 1.5-T or 3-T machines according to each institutional AS practice [11]. The imaging protocol comprised multiplanar T2-weighted images and multiple *b*-value diffusion weighting for the creation of the apparent diffusion coefficient map, along with a dedicated high *b*-value sequence of at least ≥ 1400 s/mm² and additional dynamic contrast-enhanced sequences in most of the centres, in line with the PI-RADS technical recommendations.

For the purposes of the study, all scans were re-reported by local expert genitourinary radiologists (reporting more than 500 prostate MR scans per year) at each participating centre between July 2021 and July 2022. Radiological change over time was assessed using the PRECISE v1 score (PRECISE 1 and 2: radiological regression; PRECISE 3 (subsequently divided into 3-nonvisible and 3-visible): radiological stability; PRECISE 4–5: radiological progression) according to changes in size, lesion score and/or lesion conspicuity as per PRECISE recommendations and blinded to any subsequent clinical outcomes.

### Outcome

The primary outcome was the association between radiological change on MRI and biopsy progression defined as an increase in Gleason score from diagnostic biopsy (i.e., from GG 1 to GG ≥ 2, or from GG 2 to GG ≥ 3), irrespective of whether the highest grading came from systematic or MRI-targeted biopsy cores, as per the original PRECISE consensus [9].

### Statistical analysis

Continuous variables were summarised using medians and interquartile ranges (IQR), with differences assessed for statistical significance via the Mann–Whitney test. Categorical variables were summarised by frequencies and percentages, and the Wilcoxon test was used to determine the statistical significance. Kaplan–Meier landmark analyses, with time zero considered as the date of first follow-up MRI (i.e., time of first PRECISE score), were carried out to estimate biopsy progression-free survival according to the first PRECISE score.

Given the significant variability of time to biopsy among patients with low PRECISE scores, the odds of progression at biopsies performed after each follow-up scan in patients who did not undergo additional MRIs before the biopsy were evaluated. Based on the median number of scans per patient in the study population, the association up to the third follow-up scan (i.e., the fourth MRI) was evaluated. For each interval, a separate multivariable logistic regression model with the PRECISE score as predictor (PRECISE 4–5 vs 1–3 as reference) and biopsy progression (higher GG compared to baseline, and thereafter as progression to GG ≥ 3) as the outcome was created. Each model was adjusted for the following covariates (age at AS entry, PSA and PSA density prior to MRI, GG on the latest biopsy, and baseline PI-RADS score). For each interval with a significant association between the PRECISE score and progression, sensitivity, specificity, positive predictive value (PPV) and negative predictive value (NPV), as well as diagnostic accuracy according to different biopsy thresholds and definitions of progression, were calculated. A further subgroup analysis to account for centres using MRI without contrast medium (biparametric MRI) was performed according to the type of first follow-up MRI (multiparametric vs biparametric), irrespective of the type of baseline scan. Statistical analyses were performed using R software (Version 4.5.1; The R Foundation for Statistical Computing).

## Results

### Clinical characteristics

**Fig. 1:**
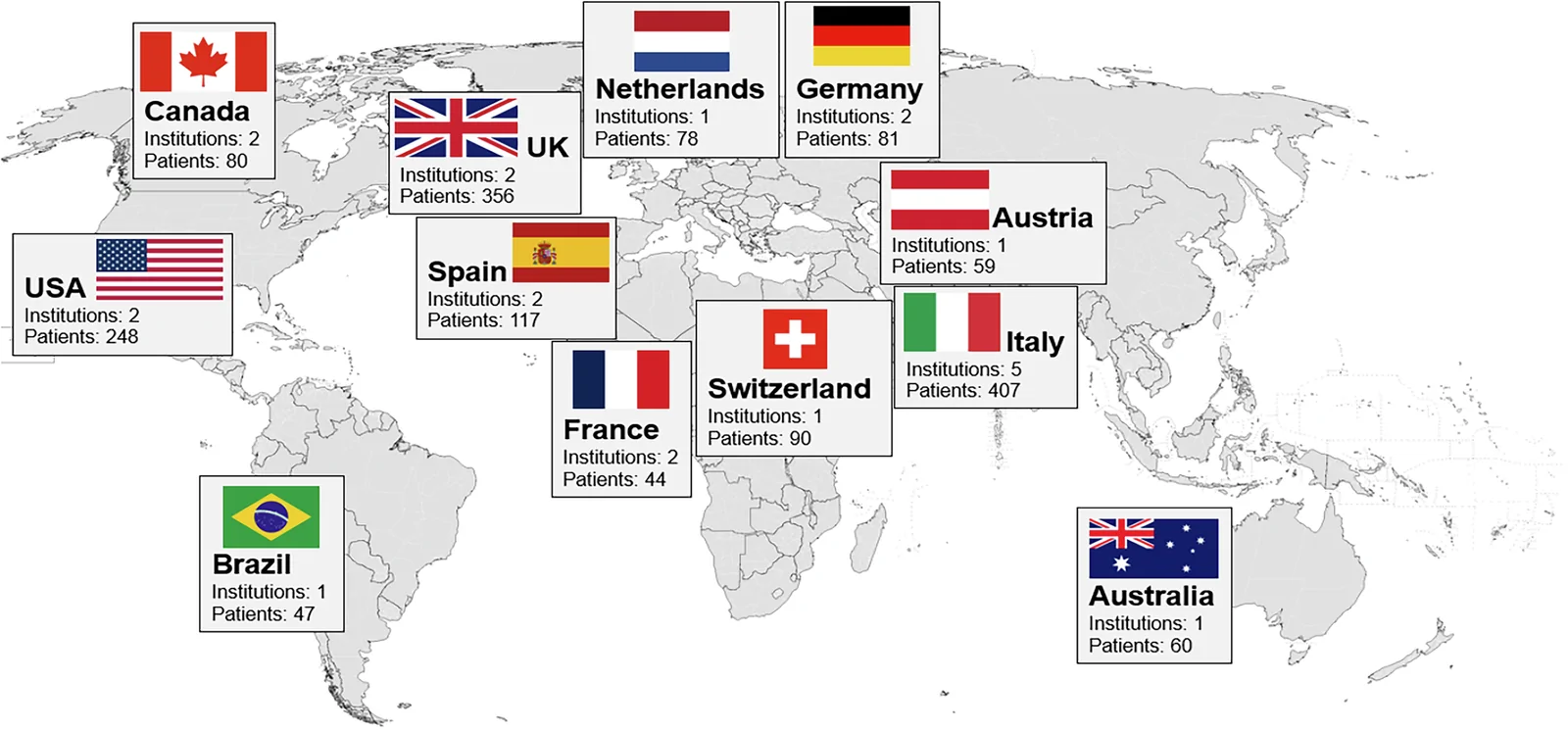
Global map of countries (*n* = 22), institutions and number of patients included in this study. UK, United Kingdom; USA, United States of America

A total of 22 centres from Europe, North and South America, and Australia participated in the study (Fig. 1) with a total of 4597 scans. Overall, 1667 patients were finally included in this study (Supplementary Table 1). Median age was 64 years (IQR: 58–68) and median baseline PSA was 5.7 ng/mL (IQR: 4.2–7.7). Median follow-up from diagnosis was 4 years (IQR: 2.1–6.3). Of 1667 men included, 1493 (90%) had GG 1 at baseline and 890 (53%) had MRI before diagnostic biopsy.

A detailed breakdown of characteristics at each institution is reported in Table 1.

**Table 1:** Relative contribution and key cohort characteristics of each institution’s patients with at least one follow-up MRI and subsequent biopsy (*n* = 1667)

Institution and country	MRI scanners	Endorectal coil	Technique	Mandated surveillance biopsies	Patients, *n* (%)	Year of first baseline MRI	Baseline PSA (ng/mL), median (IQR)	No. of biopsies, median (IQR)	No. of scans, median (IQR)	Follow-up, (years), median (IQR)
University College London (UK)	1.5–3 T	-	Multiparametric	-	230 (14)	2005	6 (4.6–8.1)	2 (2–3)	4 (3–6)	8.8 (5.9–11.1)
San Raffaele University Hospital (Italy)	1.5 T	Yes	Multiparametric	Yes	188 (11)	2010	6 (4.5–7.9)	2 (2–3)	2 (2–3)	2.7 (1.7–5.1)
Essen University Hospital (Germany)	1.5–3 T	-	Multiparametric	Yes	30 (1.8)	2015	6.2 (5.4–7)	2 (2–2)	2 (2–2)	1.8 (1.0–2.6)
Pitié Salpétrière-(France)	1.5–3 T	-	Multiparametric	Yes	31 (1.9)	2014	7.3 (5.5–10)	2 (2–3)	3 (2–4)	4.3 (3.2–5.6)
Fundació Puigvert (Spain)	3 T	-	Multiparametric	Yes	82 (4.9)	2015	6.1 (4.6–8.2)	2 (2–3)	3 (2–4)	4 (2.2–5.8)
Sapienza University (Italy)	1.5–3 T	Prior to 2016	Multiparametric	-	123 (7.4)	2012	6 (4.1–8)	2 (2–2)	2 (2–3)	2.1 (1.4–3.5)
San Luigi Gonzaga University Hospital (Italy)	1.5 T	Yes	Multiparametric, biparametric	Yes	40 (2.4)	2014	7 (4.7–8.7)	3 (2–3)	3 (2–4)	5 (2.3–6.7)
San Giovanni Battista Hospital (Italy)	1.5 T	-	Multiparametric	Yes	24 (1.4)	2016	6.1 (5–7.7)	2 (2–2)	2 (2–3)	2.7 (2.1–3.5)
Lausanne University Hospital (Switzerland)	3 T	Yes	Multiparametric	Yes	90 (5.4)	2008	6.3 (4.9–8.8)	3 (2–4)	4 (3–5)	4.1 (2.3–8.4)
Düsseldorf University Hospital (Germany)	3 T	-	Multiparametric, biparametric	Yes	51 (3.1)	2011	6.1 (4.7–8.6)	2 (2–3)	2 (2–3)	2 (1–3)
Addenbrooke’s Hospital Cambridge (UK)	1.5–3 T	-	Multiparametric, biparametric	Yes (only high-risk patients)	126 (7.6)	2013	6.2 (4.1–8.4)	2 (2–2)	4 (3–5)	6.7 (4.7–7.6)
Monash University (Australia)	3 T	-	Multiparametric	-	60 (3.6)	2013	5 (4–1.6.2)	2 (2–2)	2 (2–3)	1.9 (1.2–3.1)
University of Ottawa (Canada)	1.5–3 T	-	Multiparametric, biparametric	Yes	16 (1)	2015	5.2 (4.5–6.9)	3 (2–3)	2 (2–3)	4.5 (1.9–6.4)
Sunnybrook (Canada)	1.5–3 T	-	Multiparametric	Yes (only confirmatory biopsy)	64 (3.8)	2007	5.6 (4.1–7.8)	4 (3–4)	2 (2–3)	8.7 (4.9–12.3)
Cleveland Clinic (USA)	3 T	-	Multiparametric	Yes	48 (2.9)	2012	4.7 (3.7–7.2)	3 (3–4)	3 (2–4)	5.6 (4.4–7.2)
Mount Sinai (USA)	3 T	-	Multiparametric	Yes	200 (12)	2012	4.2 (2.8–5.3)	2 (2–2)	2 (2–2)	4.1 (2.4–5.9)
La Croix Du Sud (France)	1.5 T	-	Multiparametric	Yes	13 (0.8)	2014	5.6 (4.6–6)	2 (2–2)	3 (2–4)	3 (1.8–5.5)
Medical University of Vienna (Austria)	3 T	-	Multiparametric	Yes	59 (3.5)	2013	5.1 (3.8–7)	2 (2–3)	2 (2–3)	2.1 (1.1–3.3)
University of Padova (Italy)	1.5 T	-	Multiparametric	Yes	32 (1.9)	2013	6 (4.1–7.8)	2 (2–3)	2 (2–3)	4.1 (2.2–4.9)
Hospital Israelita Albert Einstein (Brazil)	1.5–3 T	-	Multiparametric	-	47 (2.8)	2010	4 (3–5.2)	2 (2–3)	3 (2–4)	3.2 (2.3–5.5)
Hospital Clínico San Carlos (Spain)	1.5–3 T	Yes	Multiparametric	Yes	35 (2.1)	2016	5.8 (4.4–6.7)	2 (2–3)	2 (2–3)	2.7 (1.8–3.9)
St. Antonius Hospital (The Netherlands)	3 T	-	Biparametric	Yes	78 (4.7)	2012	6.9 (5.3–8.3)	2 (2–3)	2 (2–2)	4.7 (3.1–5.9)
Total					1667	2005	5.7 (4.2–7.7)	2 (2–3)	2 (2–4)	4 (2.1–6.3)

Clinical and pathological characteristics at baseline, in the complete cohort of 1667 patients, are reported in Table 2. On the first MRI, 538 (32%) had PI-RADS 1–2, 350 (21%) PI-RADS 3, 638 (38%) PI-RADS 4, and 141 (9%) PI-RADS 5. Median time between baseline and first follow-up MRI was 13 months (IQR: 11–20 months). There was a median number (including baseline MRI) of 2 (IQR: 2–4) scans per patient. A total of 800 (48%) patients had three or more scans. The median number of biopsies (including diagnostic and first follow-up biopsy) was 2 (IQR: 2–3). Overall, 539 (32%) patients met the primary definition of biopsy progression.

**Table 2:** Patients and disease characteristics at baseline

Characteristic	PI-RADS 1–2	PI-RADS 3	PI-RADS 4	PI-RADS 5
Patients, *n* (% on 1667)	538 (32)	350 (21)	638 (38)	141 (9)
Age (years), median (IQR)	63 (57–68)	64 (58–69)	65 (59–69)	64 (59–69)
PSA (ng/mL), median (IQR)	5.8 (4.2–7.9)	5.3 (4–7.5)	5.7 (4.2–7.5)	5.8 (4.5–7.8)
Prostate volume (cc), median (IQR)	49 (35–68)	45 (34–65)	45 (33–60)	40 (31–55)
PSA density (ng/mL/cc), median (IQR)	0.12 (0.08–0.16)	0.12 (0.08–0.16)	0.13 (0.09–0.18)	0.15 (0.10–0.20)
Max. diameter of index lesion* (mm), median (IQR), in *n* = 1487	-	8 (6–11)	9 (7–12)	15 (11–18)
Biopsy route, *n* (%)				
Transrectal	450 (84)	273 (78)	497 (78)	104 (74)
Transperineal	88 (16)	77 (22)	141 (22)	37 (26)
Biopsy method, *n* (%)				
Systematic	474 (88)	209 (60)	352 (55)	67 (48)
MRI targeted	64 (12)	141 (40)	286 (45)	74 (52)
Grade group at diagnosis, *n* (%)				
GG 1	493 (92)	326 (93)	563 (88)	111 (79)
GG 2	45 (8)	24 (7)	75 (12)	30 (21)

*PI-RADS* Prostate Imaging Reporting and Data System, *IQR* interquartile range, *PSA* prostate-specific antigen, *MRI* magnetic resonance imaging, *GG* grade group

* Defined as either the largest between two lesions with the same score, or the lesion with the highest score

### Radiological changes at first and second follow-up MRI

Table 3 reports the clinical and pathological characteristics at first follow-up MRI stratified by PRECISE score. A total of 1039 (62%) patients showed no radiological change (i.e., PRECISE 3), 186 (11%) patients showed radiological regression (i.e., PRECISE 1–2), while 442 (27%) showed progression (i.e., PRECISE 4–5). A total of 1248 (75%) patients underwent a second (after baseline biopsy) biopsy directly after the first follow-up MRI at a median time of 2 months (IQR 1–5) and were considered for measuring PRECISE score performance metrics. Among those, 300 (24%) had biopsy progression to GG ≥ 2 and 77 (6%) to GG ≥ 3 as reported in Table 4.

**Table 3:** Clinical and pathological characteristics at first follow-up MRI in 1667 patients

Characteristic	PRECISE 1	PRECISE 2	PRECISE 3	PRECISE 4	PRECISE 5
Patients	69 (4)	117 (7)	1039 (62)	416 (25)	26 (2)
Age (years), median (IQR)	63 (59–69)	65 (60–69)	65 (60–70)	66 (60–71)	70 (66–74)
PSA (ng/mL), median (IQR)	5.6 (3.9–8.0)	4.9 (3.3–6.9)	5.9 (4.2–8)	7.0 (5.1–9.4)	8.4 (6.5–9.9)
Prostate volume (cc), median (IQR)	52 (38–70)	49 (38–73)	49 (36–68)	45 (34–63)	47 (40–55)
PSA density (ng/mL/cc), median (IQR)	0.11 (0.07–0.15)	0.10 (0.06–0.13)	0.12 (0.08–0.17)	0.15 (0.10–0.22)	0.16 (0.12–0.21)
Number of lesions, *n* (%)					
1	-	50 (43)	503 (48)	304 (73)	20 (77)
2		5 (4)	100 (10)	92 (22)	5 (19)
3		2 (2)	10 (1)	13 (3)	1 (4)
Max. diameter of index lesion* (mm), median (IQR)	-	8.5 (6.7–11.0)	10 (7–12)	11 (7.4–14.0)	16 (13.0–23.0)
Baseline PI-RADS score					
PI-RADS 1–2	-	-	412 (40)	126 (30)	-
PI-RADS 3	26 (38)	49 (42)	179 (17)	92 (22)	4 (15)
PI-RADS 4–5	43 (62)	68 (58)	448 (43)	198 (48)	22 (85)
Latest biopsy GG					
No cancer	8 (12)	7 (6)	86 (8)	39 (9)	2 (8)
GG 1	53 (77)	98 (84)	839 (81)	326 (78)	20 (77)
GG ≥ 2	8 (12)	12 (10)	114 (11)	51 (12)	4 (15)

*PRECISE* prostate cancer radiological estimation of change in sequential evaluation, *IQR* interquartile range, *PSA* prostate-specific antigen, *MRI* magnetic resonance imaging, *PI-RADS* Prostate Imaging Reporting and Data System, *GG* grade group

* Defined as either the largest between two lesions with the same score, or the lesion with the highest score

**Table 4:** Proportion of patients meeting each definition of progression at first biopsy after each follow-up scan, according to PRECISE score, in those who underwent immediate biopsy without interim scans

	No. progressed, *n* (%)	PRECISE 1	PRECISE 2	PRECISE 3	PRECISE 4	PRECISE 5
First follow-up MRI (*n* = 1248)		51 (4)	97 (8)	729 (58)	343 (28)	26 (2)
GG ≥ 2	300 (24)	5 (9)	10 (10)	114 (16)	155 (45)	16 (62)
GG ≥ 3	77 (6)	2 (4)	1 (1)	26 (4)	40 (12)	8 (31)
Second follow-up MRI (*n* = 363)		10 (3)	15 (4)	185 (51)	145 (40)	8 (2)
GG ≥ 2	126 (35)	2 (20)	2 (13)	40 (22)	78 (54)	4 (50)
GG ≥ 3	23 (6)	1 (10)	-	4 (2)	17 (12)	1 (12)
Third follow-up MRI (*n* = 164)		2 (1)	7 (4)	82 (50)	68 (42)	5 (3)
GG ≥ 2	66 (40)	-	1 (14)	25 (30)	37 (54)	3 (60)
GG ≥ 3	18 (11)	-	1 (14)	5 (6)	10 (15)	2 (40)

*PRECISE* prostate cancer radiological estimation of change in sequential evaluation, *MRI* magnetic resonance imaging, *GG* grade group

Supplementary Table 2 shows the rates of progression according to the PRECISE score and adjusted ORs for progression. Patients with radiological progression (PRECISE 4–5) had 4.53-fold increased odds of biopsy progression compared to those with signs of regression or stability (PRECISE 1–3) (95% CI: 3.37–6.12; *p* < 0.001). After the first follow-up MRI, the 2- and 5-year progression-free survival rates for those with PRECISE 4–5 were 49% (95% CI: 44–54%) and 35% (95% CI: 29–42%), respectively.

**Fig. 2:**
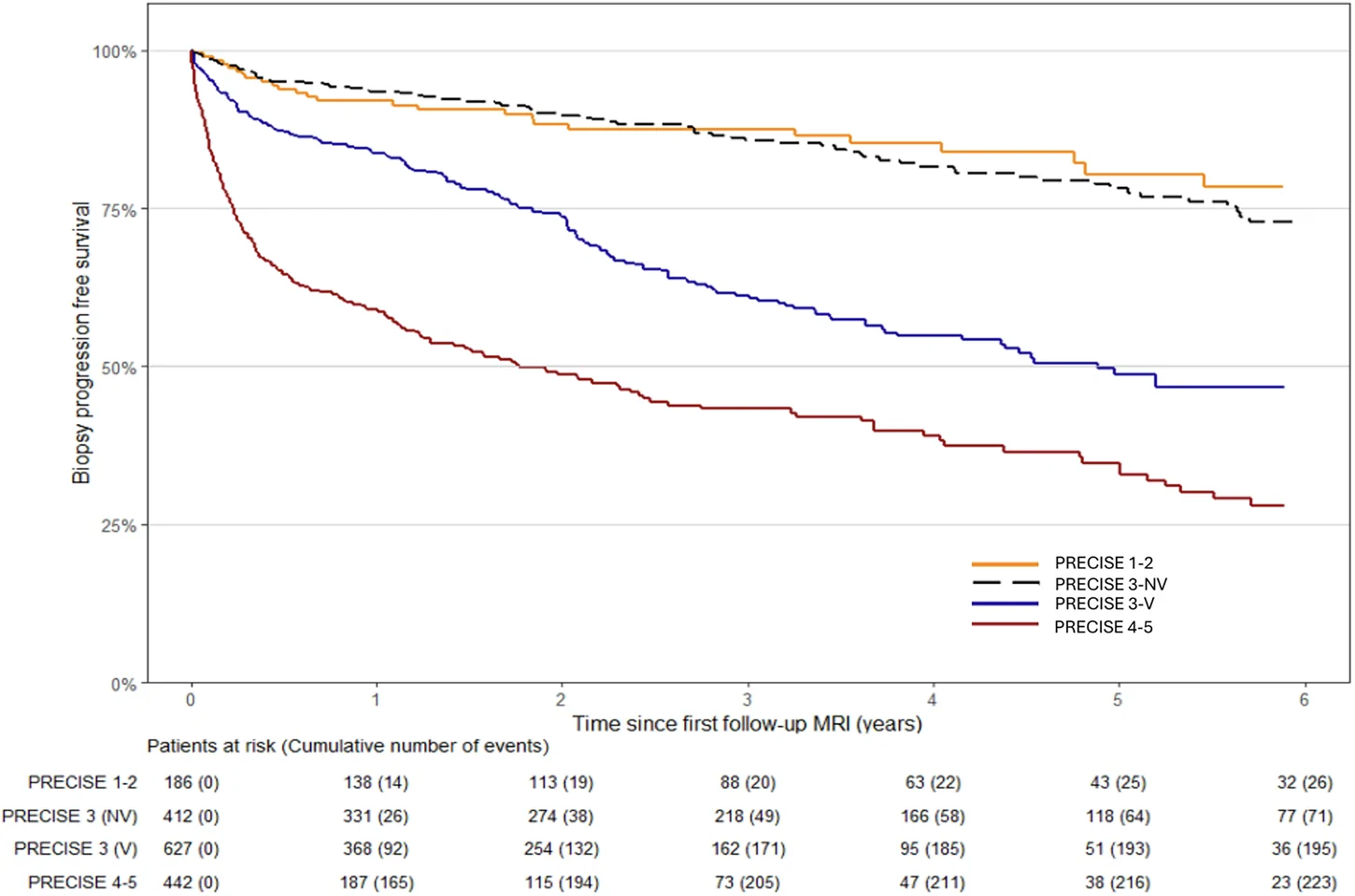
Kaplan–Meier estimates of freedom from overall biopsy progression (i.e., higher-grade group compared to baseline) according to the first MRI-derived PRECISE score in the primary study population (*n* = 1667). Time starts at the first follow-up MRI and approximates 1–2 years after the initiation of active surveillance. Patients who progressed before the first follow-up MRI were included in this analysis, and time to progression was calculated from the date of the first follow-up MRI. There were three patients who progressed before the first follow-up MRI, and whose further biopsies showed Grade Group 1 prostate cancer. Since we found an association between the baseline MRI suspicion score and MRI changes at first follow-up MRI, we stratified patients with stable findings (i.e., PRECISE 3) according to baseline MRI report. The solid yellow line represents patients with PRECISE 1–2, while the black dashed line represents patients with no visible lesion at baseline MRI, who remained stable at first follow-up MRI (PRECISE 3 (NV)). The blue and red solid lines represent patients with visible lesions at baseline MRI who remained stable at first follow-up MRI (PRECISE 3 (V)), and those who had signs of radiological progression (PRECISE 4–5), respectively

For patients with stable MRI (PRECISE 3), those without visible lesions (PRECISE 3-NonV) at baseline had progression-free rates much closer to those with PRECISE scores of 1 and 2 (90% and 78% at 2 and 5 years, respectively). The 2- and 5-year progression-free survival rates for patients with stable MRI findings and visible lesions (PRECISE 3-V) at baseline MRI were 74% and 49%, respectively (Fig. 2).

### Radiological changes at the third and fourth follow-up MRI

A total of 800 patients (48%) underwent a third scan at a median follow-up of 33 months (95% CI: 25–46), and 580 (72%) of them received a subsequent biopsy. After excluding 217 (37%) patients who underwent an interim MRI, 363 (63%), at a median time of 3 months (IQR 1–5), were available for evaluating the impact of the PRECISE score. Overall, 153/363 (42%) patients had a PRECISE score of 4–5, and 126/363 (35%) had biopsy progression. Patients with radiological progression (PRECISE 4–5) at second follow-up MRI had 4.19-fold increased odds of biopsy progression compared to patients with stability or regression (95% CI: 2.53–7.04; *p* < 0.001).

We also observed an association between radiological and biopsy progression in 164 patients who underwent a fourth MRI followed by a biopsy without in-between scans with an odds ratio of 2.42 (95% CI: 1.15–5.17; *p* = 0.02). The median time to the fourth scan was 50 months (IQR: 40–64).

### Performance of the PRECISE score and assessment of clinical utility

Performance metrics of the PRECISE score at each follow-up MRI are reported in Table 5. Using a threshold for a positive PRECISE score for progression being PRECISE ≥ 4 sensitivity, specificity, PPV and NPV for the primary definition of progression were 57%, 79%, 46%, and 85% for the first follow-up scan, 65%, 70%, 54%, and 79% for the second follow-up scan and 61%, 66%, 55% and 71% for the third follow-up scan. Using a lower threshold for a positive PRECISE score (PRECISE ≥ 3), we observed the following sensitivity, specificity, PPV and NPV: (1) 95%, 14%, 26%, 90% at first follow-up MRI; (2) 97%, 9%, 36%, and 84%, at second follow-up MRI and (3) 98%, 8%, 42% and 89% at third follow-up MRI.

**Table 5:** Overall sensitivity, specificity, positive- and negative predictive value, and accuracy, with 95% confidence intervals, for each threshold of MRI change at each follow-up MRI, respectively

MRI change threshold	Progression	Sensitivity	Specificity	PPV	NPV	Accuracy
First follow-up MRI						
PRECISE ≥ 4	GG ≥ 2	0.57 (0.51-0.63)	0.79 (0.76-0.82)	0.46 (0.41-0.52)	0.85 (0.83-0.88)	0.74 (0.71-0.76)
	GG ≥ 3	0.62 (0.51-0.73)	0.73 (0.70-0.75)	0.13 (0.10-0.17)	0.97 (0.95-0.98)	0.72 (0.69-0.74)
PRECISE ≥ 3 (V)^a^	GG ≥ 2	0.87 (0.83-0.91)	0.40 (0.37-0.43)	0.31 (0.28-0.35)	0.91 (0.87-0.93)	0.51 (0.48-0.54)
	GG ≥ 3	0.87 (0.77-0.94)	0.35 (0.32-0.38)	0.08 (0.06-0.10)	0.98 (0.96-0.99)	0.38 (0.35-0.41)
PRECISE ≥ 3^b^	GG ≥ 2	0.95 (0.92-0.97)	0.14 (0.12-0.17)	0.26 (0.23-0.29)	0.90 (0.84-0.94)	0.34 (0.31-0.36)
	GG ≥ 3	0.96 (0.89-0.99)	0.13 (0.11-0.15)	0.07 (0.05-0.08)	0.98 (0.94-0.99)	0.18 (0.16-0.20)
PRECISE 2, 3 (V)^a^, 4-5	GG ≥ 2	0.90 (0.86-0.93)	0.31 (0.28-0.34)	0.29 (0.26-0.32)	0.91 (0.87-0.94)	0.45 (0.42-0.48)
	GG ≥ 3	0.88 (0.79-0.95)	0.27 (0.24-0.29)	0.07 (0.06-0.09)	0.97 (0.95-0.99)	0.30 (0.28-0.33)
Second follow-up MRI						
PRECISE ≥ 4	GG ≥ 2	0.65 (0.56-0.73)	0.70 (0.64-0.76)	0.54 (0.45-0.62)	0.79 (0.73-0.84)	0.68 (0.63-0.73)
	GG ≥ 3	0.78 (0.56-0.93)	0.60 (0.55-0.66)	0.12 (0.07-0.18)	0.98 (0.95-0.99)	0.61 (0.56-0.66)
PRECISE ≥ 3 (V)	GG ≥ 2	0.84 (0.77-0.90)	0.43 (0.37-0.50)	0.44 (0.38-0.51)	0.84 (0.76-0.90)	0.58 (0.52-0.63)
	GG ≥ 3	0.96 (0.78-0.99)	0.36 (0.31-0.41)	0.09 (0.06-0.14)	0.99 (0.96-0.99)	0.40 (0.35-0.45)
PRECISE ≥ 3	GG ≥ 2	0.97 (0.92-0.99)	0.09 (0.06-0.13)	0.36 (0.31-0.41)	0.84 (0.64-0.95)	0.39 (0.34-0.45)
	GG ≥ 3	0.96 (0.78-0.99)	0.07 (0.05-0.10)	0.07 (0.04-0.10)	0.96 (0.80-0.99)	0.13 (0.09-0.17)
PRECISE 2, 3 (V)^a^, 4-5	GG ≥ 2	0.86 (0.78-0.91)	0.38 (0.32-0.44)	0.42 (0.36-0.49)	0.83 (0.75-0.90)	0.55 (0.49-0.60)
	GG ≥ 3	0.96 (0.79-0.99)	0.31 (0.27-0.37)	0.09 (0.05-0.13)	0.99 (0.95-0.99)	0.36 (0.31-0.41)
Third follow-up MRI						
PRECISE ≥ 4	GG ≥ 2	0.61 (0.48-0.72)	0.66 (0.56-0.76)	0.55 (0.43-0.66)	0.71 (0.61-0.80)	0.64 (0.56-0.71)
	GG ≥ 3	0.67 (0.41-0.87)	0.58 (0.50-0.66)	0.16 (0.09-0.27)	0.93 (0.86-0.98)	0.59 (0.51-0.67)
PRECISE ≥ 3 (V)	GG ≥ 2	0.85 (0.74-0.92)	0.46 (0.36-0.56)	0.51 (0.42-0.61)	0.82 (0.69-0.91)	0.62 (0.54-0.69)
	GG ≥ 3	0.72 (0.47-0.90)	0.34 (0.27-0.43)	0.12 (0.07-0.20)	0.91 (0.80-0.97)	0.38 (0.31-0.46)
PRECISE ≥ 3	GG ≥ 2	0.98 (0.92-0.99)	0.08 (0.04-0.15)	0.42 (0.34-0.50)	0.89 (0.52-0.99)	0.45 (0.37-0.52)
	GG ≥ 3	0.94 (0.73-0.99)	0.05 (0.02-0.11)	0.11 (0.07-0.17)	0.89 (0.52-0.99)	0.15 (0.10-0.22)
PRECISE 2, 3 (V)^a^, 4-5	GG ≥ 2	0.86 (0.76-0.94)	0.40 (0.30-0.50)	0.49 (0.40-0.59)	0.81 (0.67-0.91)	0.59 (0.51-0.66)
	GG ≥ 3	0.78 (0.52-0.94)	0.30 (0.23-0.38)	0.12 (0.07-0.19)	0.92 (0.80-0.98)	0.35 (0.28-0.43)

*MRI* magnetic resonance imaging, *PPV* positive predictive value, *NPV* negative predictive value, *PRECISE* prostate cancer radiological estimation of change in sequential evaluation, *GG* grade group

^a^ PRECISE 3 (V) denotes a stable MR scan with a visible lesion

^b^ PRECISE 3 includes stable MR scans (visible and non-visible lesions)

A very high NPV was observed for each threshold for progression to GG ≥ 3. If an AS protocol mandated a repeat biopsy based only on signs of MRI progression, 70% of patients would avoid biopsy, and the risk of GG ≥ 2 would be 15% (CI: 12–27%) at first follow-up scan. If a repeat biopsy of visible disease had been performed in all patients with stable findings (i.e., PI-RADS ≥ 4 at baseline and follow-up MRIs), as well as those with progression, slightly more than 30% of patients would avoid a biopsy, and 9–18% of progressions would be missed.

Overall diagnostic accuracy varied significantly according to each threshold for progression, being globally high (74%, 95% CI: 71–76%) for the stringent definition of progression as PRECISE 4–5, and decreased according to whether lower PRECISE tiers were included within progressions (Table 5).

We did not observe extreme variations of performance metrics according to whether the first MRI-derived PRECISE score was generated from a biparametric or multiparametric scan, as reported in Supplementary Table 3. In particular, we observed a slightly lower NPV and accuracy of biparametric vs multiparametric MRI for each PRECISE threshold; however, such variation with wide confidence intervals is plausibly explained by the small number of biparametric scans (*n* = 47 vs *n* = 1201).

**Fig. 3:**
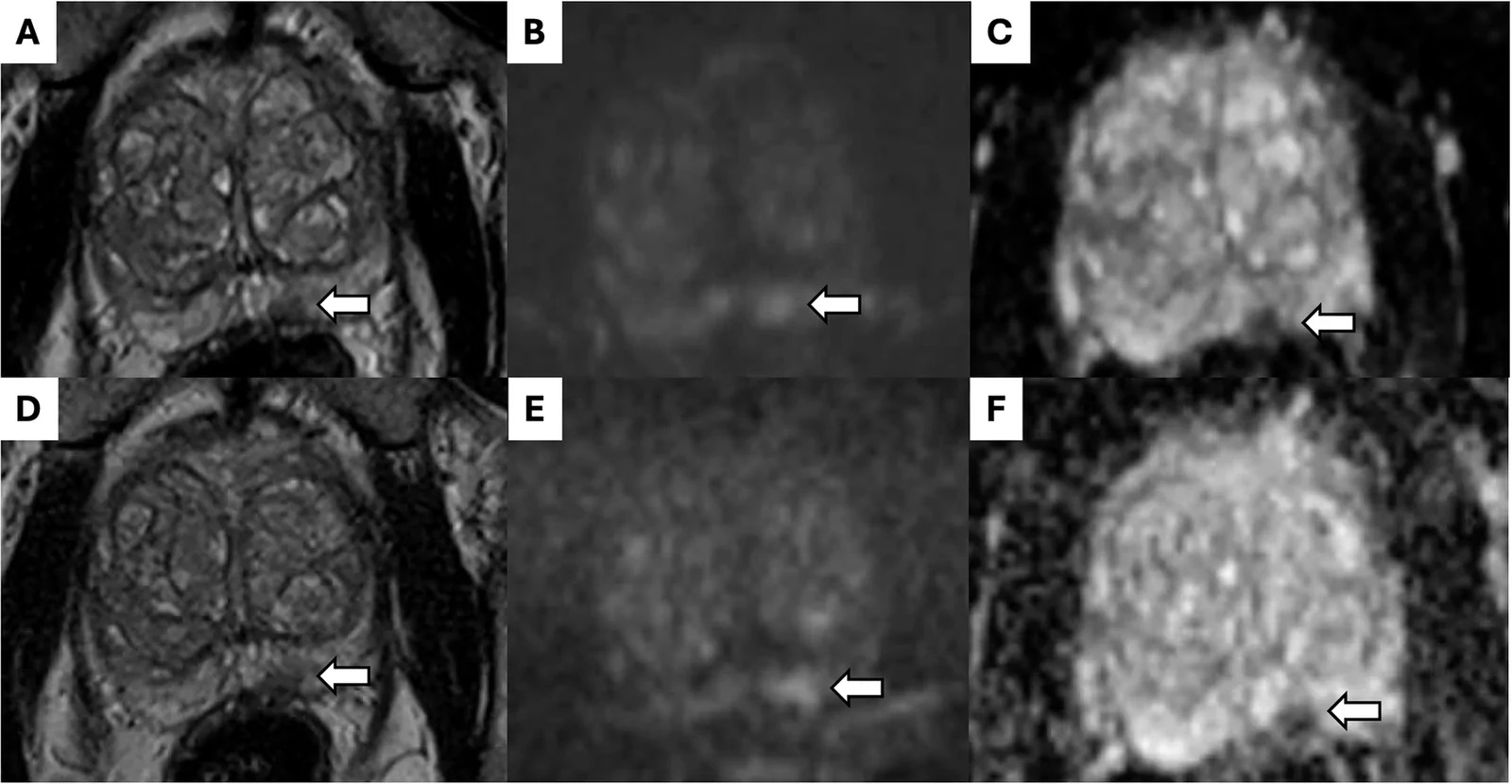
Stable PI-RADS 4 lesion (arrows) in the left mid-gland peripheral zone on T2-WI (**A**, **D**), high *b*-value (**B**, **E**) and ADC map (**C**, **F**) at baseline (**A**–**C**) and after 1 year (**D**–**F**) in a 70-year-old patient. Biopsy confirmed Gleason Grade Group 1. PRECISE score: 3-V

**Fig. 4:**
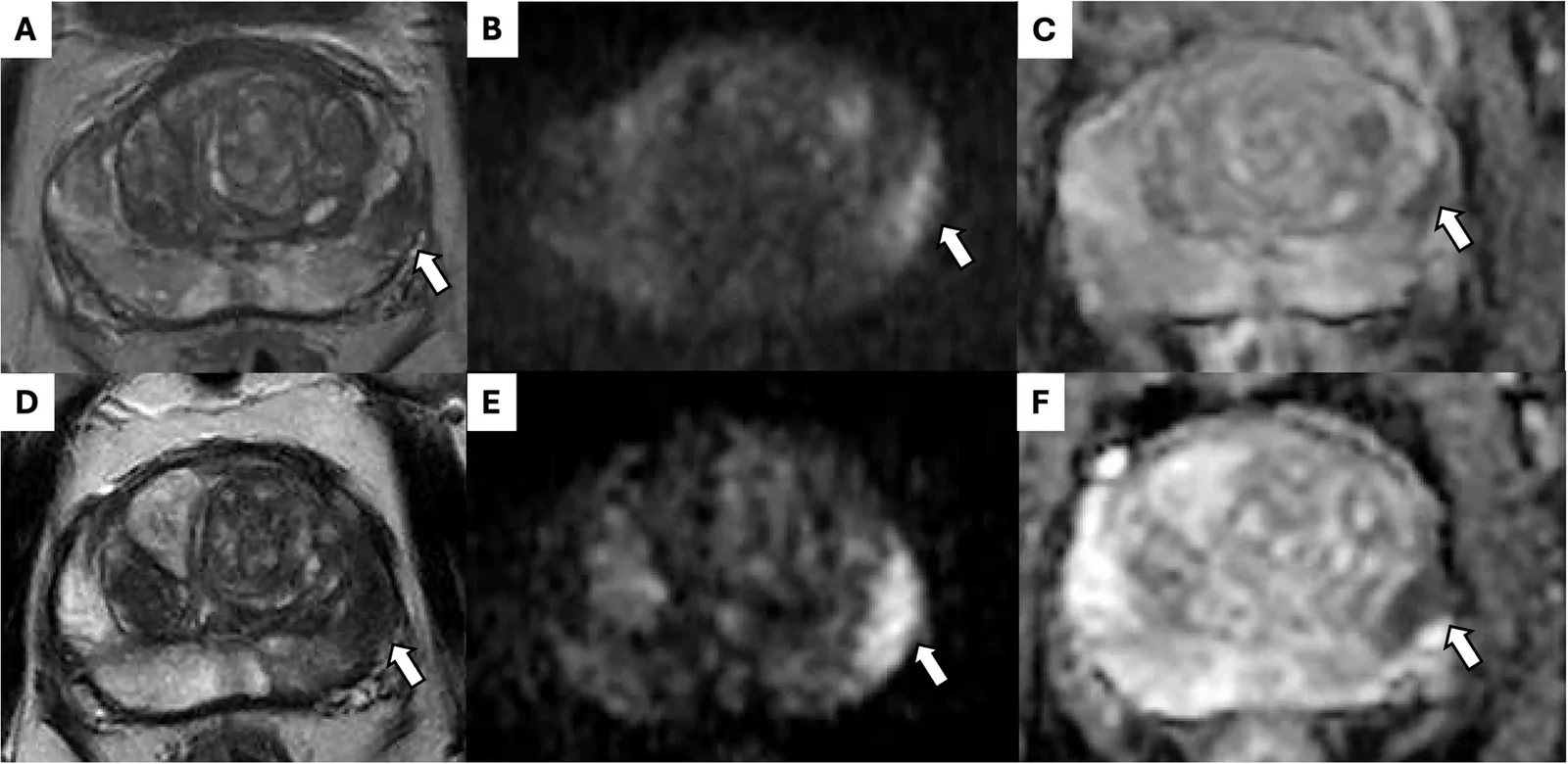
Enlarging PI-RADS 4 lesion (arrows) in the left posterolateral PZ at mid-gland on T2-WI (**A**, **D**), high *b*-value (**B**, **E**) and ADC map (**C**, **F**) at baseline (**A**–**C**) and after 1 year (**D**–**F**) in a 76-year-old patient. Initial targeted biopsy revealed Gleason Grade Group 1. At follow-up MRI, the lesion showed an increase in size and conspicuity and targeted biopsy showed Gleason Grade Group 2. PRECISE score: 4

A case of radiological stability (PRECISE 3-V) and a case of radiological progression (PRECISE 4) are reported in Fig. 3 and Fig. 4, respectively.

## Discussion

Our multicentre international study assessed the PRECISE score’s utility for prostate cancer on AS, focusing on its association with biopsy progression. We found that 42% of patients showed radiological progression (PRECISE 4–5), 11% regression (PRECISE 1–2), and the remainder radiological stability (PRECISE 3). Patients with radiological progression (PRECISE 4–5) had significantly increased odds of biopsy progression compared to those with stable or regressing MRI findings (PRECISE 1–3). Their 2- and 5-year progression-free survival rates were 49% and 35%, respectively, highlighting the clinical significance of radiological progression. These findings align with the PROMM-AS study [12], in which the PRECISE scores strongly predicted histological progression (PPV 81%, NPV 88% for GG 1; PPV 92%, NPV 50% for GG 2, respectively). Similarly to our results, the PROMM-AS study demonstrated that when MRI findings remained stable (i.e., PRECISE 1–3), the NPV for ruling out GG reclassification was 88% over a 2-year period, highlighting the utility of MRI stability for identifying patients at low risk of progression. Taken together, our results corroborate PROMM-AS, showing that radiological progression reflects clinically meaningful risk and that MRI, especially stable findings, is a valuable tool for monitoring patients under AS.

Our analysis further distinguished stable MRI findings (PRECISE 3) by baseline lesion visibility, as recommended by the PRECISE v2 criteria [10]. Patients with PRECISE 3-NonV at baseline showed progression-free rates similar to PRECISE 1–2. Conversely, those with PRECISE 3-V had 2- and 5-year progression-free survival rates of 74% and 49%, respectively. We also noted that an AS protocol relying solely on MRI progression (PRECISE ≥ 4 cutoff to perform a follow-up biopsy) could avoid biopsy in 70% of patients, with a 15% risk of missing GG ≥ 2 progression at first follow-up biopsy.

The 27% radiological progression rate in our cohort underscores prostate cancer’s dynamic nature under AS [13]. The strong link between radiological progression (PRECISE 4–5) and subsequent biopsy progression to higher GGs validates PRECISE as a clinically meaningful indicator of disease reclassification.

The distinction based on baseline lesion visibility (PRECISE 3-NonV vs PRECISE 3-V) shows that PRECISE 3-NonV patients appear to have a more indolent course, similar to those with radiological regression (PRECISE 1–2), suggesting potential for de-escalated follow-up withholding scheduled biopsies. However, PRECISE 3-V patients, despite radiological stability, require continued vigilance due to higher progression risk.

The PRECISE score’s high NPV, especially for GG ≥ 3 progression, indicates its strong ability to rule out higher-grade disease. This supports MRI-led protocols for reducing unnecessary biopsies, as shown by the 70% biopsy avoidance rate with a PRECISE ≥ 4 cutoff (i.e., performing a follow-up biopsy only for PRECISE 4–5). Yet, the trade-off between biopsy avoidance and missed progressions (9–18% for GG ≥ 2) highlights the ongoing challenge of balancing patient burden with safety. Lower sensitivity and PPV at the PRECISE ≥ 4 threshold means some biopsy progressions might be missed, necessitating careful clinical considerations.

Our findings align with existing literature on MRI and PRECISE in AS, as the strong association between PRECISE 4–5 and biopsy progression to higher GG is a consistent theme [14]. Our study extends this by quantifying progression-free survival rates for PRECISE 4–5 patients.

The PRECISE v2 recommendations, which subdivided PRECISE 3 into 3-NonV and 3-V, are directly supported by our data on differential progression-free survival rates, validating the clinical relevance of this distinction [10].

Our PRECISE score performance metrics, particularly high NPVs for ruling out GG ≥ 3 progression, align with a meta-analysis by Rajwa et al [14]. While they cautioned against relying solely on MRI, our multicentre data highlights a significant 70% reduction in unnecessary biopsies using a PRECISE ≥ 4 cutoff; however, this must be balanced against the 15% risk of missing GG ≥ 2 disease. Rather than advocating for MRI as a solitary metric, these data quantify the clinical trade-offs inherent in reducing biopsy frequency during AS. Given significant variations in PPV and NPV according to whether the progression definition was stringent (i.e., PRECISE 4–5) or broader (i.e., PRECISE ≥ 3), we also observed relevant variations in overall accuracy. In particular, for the high NPV of 91% for radiological progression defined as PRECISE 4–5 and visible PRECISE 3, accuracy was lower (51%) compared to the higher accuracy (74%) for the stringent PRECISE 4–5 definition, which was however associated with a lower NPV (85%). This warrants clinicians to use caution, as higher thresholds for progression are associated with a higher risk of missing GG ≥ 2 disease, while broader thresholds increase the risk of unnecessary biopsies.

Englman et al have recently published the updated results of their large cohort on MRI-led risk-adapted AS that further supports the importance of MRI visibility at baseline and during follow-up [7]. Their findings that MRI visibility and secondary Gleason pattern 4 at baseline are associated with progression to treatment and higher-grade disease align with the observation that PRECISE 3-V patients have a higher progression risk than PRECISE 3-NonV patients in a multicentre setting.

Our study offers several key strengths. First, it is a pioneering pilot multicentre international study on progression outcomes in AS, heavily relying on MRI. The inclusion of both academic and non-academic institutions enhances the generalisability of our results to a broader clinical landscape [15]. Second, our cohort included both low and favourable intermediate-risk prostate cancer patients, providing a comprehensive understanding of PRECISE utility across different subtypes. Third, our outcomes compare favourably to single-centre studies, further validating our findings’ robustness in a multicentre framework.

Despite its strengths, our study has limitations. Its retrospective design introduces potential biases. Although there was no central image review, local radiologists were highly experienced. However, we believe that this variability, while a limitation for strict standardisation, strengthens generalisability by reflecting heterogeneous clinical practices and by representing a real-world scenario.

A second limitation is the ascertainment bias, as urologists’ decisions on biopsy thresholds, particularly for higher PI-RADS scores or PSA density with stable MRI, influenced outcomes [16]. This clinical discretion, though real-world, could impact observed biopsy progression rates. We chose to select only imaging and biopsy intervals resembling those of strict follow-up protocols to mitigate such bias; in fact, we evaluated only biopsies after each given follow-up MRI, without in-between scans. Also, a substantial proportion of patients underwent systematic biopsy, which served as the reference standard, potentially affecting the accuracy compared with targeted biopsy.

Another limitation is the lack of a predefined, standardised protocol for the evaluation of radiologic progression across participating centres; this is particularly significant given recent evidence demonstrating high inter- and intra-rater variability in MRI-based lesion measurements during AS [17].

An additional limitation of our study is that the exclusion of low-quality scans was based on subjective assessment by local radiologists. Consequently, some observed radiologic progression may reflect improvements in image quality rather than true biological change.

Lastly, baseline differences in inclusion criteria across centres led to cohort variations. However, this heterogeneity is also a reflection of the current clinical reality, as there are no universally standardised AS guidelines with specific inclusion and exclusion criteria across all institutions [2]. In our opinion, this “real-world scenario” context enhances the applicability of our findings to various clinical settings.

In conclusion, our findings have important clinical implications for AS. For patients with radiological regression (PRECISE 1–2) and stable non-visible MRI findings (PRECISE 3-NonV), biopsy progression is infrequent, with very low long-term progression rates. This suggests potential for de-escalating follow-up, including reducing biopsy frequency, for these subgroups, minimising patient burden safely. However, guiding biopsy decisions with PRECISE requires careful threshold consideration. While high NPV for GG ≥ 3 progression was consistent, the NPV for a broader threshold (biopsy for stable visible findings and progression) decreased at the second follow-up MRI, highlighting the dynamic nature of AS [13].

Future research must focus on establishing a universal, appropriate threshold (e.g., tumour size/volume) for safely omitting biopsy in patients with stable radiological findings. Investigating the combination of MRI with other clinical parameters (PSA density, prior MRI/biopsy history) could guide avoiding biopsies when scans are reassuring [18]. In particular, we did not focus on prediction, as our objective was to evaluate the PRECISE scoring system in a multi-institutional environment. Further research is ongoing to elucidate whether biopsy avoidance can be guided by PSA density in addition to MRI appearance. Once this threshold is established, understanding when follow-up MRI frequency itself can be safely delayed without missing significant progression will be crucial. These efforts are vital for optimising AS protocols, balancing patient quality of life with oncological control.

## Supplementary information

ELECTRONIC SUPPLEMENTARY MATERIAL

## Abbreviations

AS: Active surveillance
GG: Gleason grade group
IQR: Interquartile range
NPV: Negative predictive value
PPV: Positive predictive value
PRECISE: Prostate cancer radiological estimation of change in sequential evaluation
PSA: Prostate-specific antigen
