# Supplementary material for: Multicentre validation of the PRECISE scoring system for prostate MRI during active surveillance
Source: Eur Radiol. 2026 May 4;36(10):7940–53. doi: 10.1007/s00330-026-12570-z (PMC13574842; doi:10.1007/s00330-026-12570-z)
Supplement: Supplementary file 1 [file 330_2026_12570_MOESM1_ESM.docx]

**Supplementary Table 1:** Number of initial patients provided and number of patients meeting the study criteria stratified by Centre.

| **Institution** | Patients provided | Patients meeting the study criteria  (% over provided pts) |
| --- | --- | --- |
| **University College London** | 340 | 230 (68) |
| **Milano San Raffaele** | 207 | 188 (91) |
| **University Hospital Essen** | 50 | 30 (60) |
| **Pitié Salpétrière-Tenon Hospital (Paris)** | 38 | 31 (82) |
| **Fundació Puigvert** | 124 | 82 (66) |
| **Sapienza University** | 156 | 123 (79) |
| **San Luigi Gonzaga University Hospital** | 58 | 40 (69) |
| **San Giovanni Battista Hospital** | 31 | 24 (77) |
| **Lausanne University Hospital** | 107 | 90 (84) |
| **Düsseldorf** | 52 | 51 (98) |
| **Addenbrooke’s Hospital (Cambridge)** | 247 | 126 (51) |
| **Monash University (Melbourne)** | 61 | 60 (98) |
| **University of Ottawa** | 29 | 16 (55) |
| **Toronto Sunnybrook** | 70 | 64 (91) |
| **Cleveland Clinic (Cleveland)** | 55 | 48 (87) |
| **Mount Sinai (New York)** | 798 | 200 (25) |
| **La Croix Du Sud (Toulouse)** | 13 | 13 (100) |
| **Medical University of Vienna** | 60 | 59 (98) |
| **University of Padova** | 48 | 32 (67) |
| **Hospital Israelita Albert Einstein (Sao Paulo)** | 48 | 47 (98) |
| **Hospital Clínico San Carlos (Madrid)** | 51 | 35 (69) |
| **St. Antonius Hospital (Utrecht)** | 82 | 78 (95) |
| **Total** | 2,725 | 1,667 |

**Supplementary Table 2:** Multivariable logistic regression models testing the association between PRECISE scores 4-5 overall biopsy progression, and progression to GG≥3, at each follow-up MRI followed by a biopsy without ad interim scans.

| **Follow-up scan** | **Patients (progressions)** | **Adjusted OR for PRECISE 4-5 (95% CI)** | ***p*** |
| --- | --- | --- | --- |
| **Primary definition of progression (GG ≥ 2)** | | | |
| **MRI 2** | **1248 (300)** | 4.53 (3.37-6.12) | <0.001 |
| **MRI 3** | **363 (126)** | 4.19 (2.53-7.04) | <0.001 |
| **MRI 4** | **164 (66)** | 2.42 (1.15-5.17) | 0.02 |
| **Progression to GG ≥ 3** | | | |
| **MRI 2** | **1248 (77)** | 3.7 (2.24-6.2) | <0.001 |
| **MRI 3** | **363 (23)** | 4.65 (1.71-14.9) | 0.005 |
| **MRI 4** | **164 (18)** | 2.24 (0.73-7.37) | 0.2 |

**Abbreviations:** OR: Odds Ratio; CI: Confidence Intervals; MRI: Magnetic Resonance Imaging; PRECISE: Prostate Cancer Radiological Estimation of Change in Sequential Evaluation.

**Supplementary Table 3:** Overall sensitivity, specificity, positive- and negative predictive value, and accuracy, with 95% confidence intervals, for each threshold of MRI change at first follow-up MRI, according to MRI protocol used at first follow-up MRI.

| MRI change threshold | Progression | Sensitivity | Specificity | PPV | NPV | Accuracy |
| --- | --- | --- | --- | --- | --- | --- |
| Multiparametric | | | | | | |
| PRECISE ≥4 | GG≥2 | 0.57 (0.51-0.63) | 0.79 (0.76-0.82) | 0.46 (0.41-0.51) | 0.86 (0.83-0.88) | 0.74 (0.71-0.76) |
|  | GG≥3 | 0.64 (0.52-0.75) | 0.73 (0.70-0.75) | 0.13 (0.10-0.17) | 0.97 (0.96-0.98) | 0.72 (0.70-0.75) |
| PRECISE ≥3 (V) ^a^ | GG≥2 | 0.87 (0.83-0.91) | 0.39 (0.36-0.42) | 0.31 (0.28-0.34) | 0.91 (0.88-0.94) | 0.50 (0.48-0.53) |
|  | GG≥3 | 0.88 (0.78-0.94) | 0.34 (0.31-0.37) | 0.08 (0.06-0.10) | 0.98 (0.96-0.99) | 0.37 (0.35-0.40) |
| PRECISE ≥3 ^b^ | GG≥2 | 0.95 (0.91-0.97) | 0.14 (0.12-0.17) | 0.25 (0.23-0.28) | 0.90 (0.84-0.94) | 0.33 (0.31-0.36) |
|  | GG≥3 | 0.96 (0.88-0.99) | 0.13 (0.11-0.15) | 0.07 (0.05-0.08) | 0.98 (0.94-0.99) | 0.18 (0.16-0.20) |
| PRECISE 2, 3 (V)^a^, 4-5 | GG≥2 | 0.91 (0.87-0.94) | 0.30 (0.27-0.33) | 0.29 (0.26-0.32) | 0.91 (0.88-0.94) | 0.44 (0.41-0.47) |
|  | GG≥3 | 0.89 (0.79-0.95) | 0.26 (0.23-0.29) | 0.07 (0.06-0.09) | 0.97 (0.95-0.99) | 0.30 (0.27-0.32) |
| Biparametric | | | | | | |
| PRECISE ≥4 | GG≥2 | 0.56 (0.30-0.80) | 0.77 (0.59-0.90) | 0.56 (0.30-0.80) | 0.77 (0.59-0.90) | 0.70 (0.55-0.83) |
|  | GG≥3 | 0.40 (0.05-0.85) | 0.67 (0.50-0.80) | 0.12 (0.02-0.38) | 0.90 (0.74-0.98) | 0.64 (0.49-0.77) |
| PRECISE ≥3 (V) | GG≥2 | 0.81 (0.54-0.96) | 0.65 (0.45-0.81) | 0.54 (0.33-0.74) | 0.87 (0.66-0.97) | 0.70 (0.55-0.83) |
|  | GG≥3 | 0.80 (0.28-0.99) | 0.52 (0.36-0.68) | 0.17 (0.05-0.37) | 0.96 (0.79-0.99) | 0.55 (0.40-0.70) |
| PRECISE ≥3 | GG≥2 | 0.99 (0.79-0.99) | 0.13 (0.04-0.30) | 0.37 (0.23-0.53) | 0.99 (0.40-0.99) | 0.43 (0.28-0.58) |
|  | GG≥3 | 0.99 (0.48-0.99) | 0.10 (0.03-0.23) | 0.12 (0.04-0.25) | 0.99 (0.40-0.99) | 0.19 (0.09-0.33) |
| PRECISE 2, 3 (V)^a^, 4-5 | GG≥2 | 0.81 (0.54-0.96) | 0.55 (0.36-0.73) | 0.48 (0.29-0.68) | 0.85 (0.62-0.97) | 0.64 (0.49-0.77) |
|  | GG≥3 | 0.80 (0.28-0.99) | 0.45 (0.30-0.61) | 0.15 (0.04-0.34) | 0.95 (0.75-0.99) | 0.49 (0.34-0.64) |
| Abbreviations: MRI: Magnetic Resonance Imaging; PPV: Positive Predictive Value; NPV: Negative Predictive Value; PRECISE: Prostate Cancer Radiological Estimation of Change in Sequential Evaluation; GG: Grade Group.    ^a^ PRECISE 3 (V) denotes a stable MR scan with a visible lesion  ^b^ PRECISE 3 includes stable MR scans (visible and non-visible lesions) | | | | | | |
